# Ring/U-Box Protein AtUSR1 Functions in Promoting Leaf Senescence Through JA Signaling Pathway in Arabidopsis

**DOI:** 10.3389/fpls.2020.608589

**Published:** 2020-12-16

**Authors:** Zenglin Zhang, Mengmeng Xu, Yongfeng Guo

**Affiliations:** Tobacco Research Institute, Chinese Academy of Agricultural Sciences, Qingdao, China

**Keywords:** MYC2, JA, leaf senescence, AtUSR1, ring/U-box

## Abstract

Leaf senescence is regulated by a large number of internal and environmental factors. Here, we report that AtUSR1 (U-box Senescence Related 1) which encodes a plant Ring/U-box protein, is involved in age-dependent and dark-induced leaf senescence in Arabidopsis. Expression of *AtUSR1* gene in leaves was up-regulated in darkness and during aging. Plants of *usr1*, an *AtUSR1* gene knock-down mutant, showed a significant delay in age-dependent and dark-induced leaf senescence and the delayed senescence phenotype was rescued when the *AtUSR1* gene was transferred back to the mutant plants. Meanwhile, overexpression of *AtUSR1* caused accelerated leaf senescence. Furthermore, the role of AtUSR1 in regulating leaf senescence is related to MYC2-mediuated jasmonic acid (JA) signaling pathway. MeJA treatments promoted the accumulation of *AtUSR1* transcripts and this expression activation was dependent on the function of MYC2, a key transcription factor in JA signaling. Dual-luciferase assay results indicated that MYC2 promoted the expression of *AtUSR1*. Overexpression of *AtUSR1* in *myc2* mutant plants showed precocious senescence, while *myc2* mutation alone caused a delay in leaf senescence, suggesting that AtUSR1 functions downstream to MYC2 in the JA signaling pathway in promoting leaf senescence.

## Introduction

As a process of programmed cell death, leaf senescence is important in plants’ development and response to environmental stresses (Guo and Gan, 2005; Kim et al., 2018). Under harsh environmental conditions, in order to complete their life cycle, stressed plants often endeavor to reallocate nutrients to reproductive organs via the process of leaf senescence (Sedigheh et al., 2011). Leaf senescence can be induced by a large number of endogenous and environmental factors including age, plant hormones, light conditions, abiotic stresses, and pathogen infection. Once senescence is initiated, significant changes in gene expression, metabolic, and physiological activities take place and the execution of leaf senescence eventually leads to programmed cell death (Quirino et al., 2000; Khanna-Chopra, 2012; Guo, 2013). During senescence, visible leaf yellowing can be observed as the result of chloroplast degeneration which is associated with drastic metabolic reprogramming including degradation of macromolecules, enhanced reactive oxygen species (ROS), and occurrence of membrane ion leakage (Ansari et al., 2014; Gan, 2018). During senescence, the expression of genes related to photosynthesis is down-regulated, whereas genes involved in senescence execution generally have increased expression. Genes encoding components of protein degradation pathways, for example, comprise a significant proportion of *Senescence Associated Genes* (*SAG*s; Guo et al., 2004; Lim et al., 2007; Gregersen et al., 2008; Zhang et al., 2010). As an essential part of protein degradation, the ubiquitin/26S proteasome pathway is not only important in targeting and degrading proteins, but also involved in regulation of the senescence process. Mutation in several components in the ubiquitin/proteasome system led to altered leaf senescence (Woo et al., 2001; So et al., 2019; Shim et al., 2020), suggesting a regulatory role of the ubiquitin pathway in leaf senescence. As a critical component of the ubiquitin pathway, E3 ubiquitin ligase is involved in polyubiquitination of target proteins and participates in various plant development processes including senescence. In rice, RING-type ubiquitin ligase GW2 acts as a positive regulator of leaf senescence by affecting chlorophyll degradation (Shim et al., 2020). Senescence-associated E3 ubiquitin ligase1/Plant U-box44 (SAUL1/PUB44) regulates dark-induced leaf senescence in Arabidopsis by affecting stability of the chloroplast-localized Abscisic acid (ABA)-responsive factor Senescence-Associated Protein (CSAP; So et al., 2019). RING-type E3 ligases SEVEN IN ABSENTIA OF ARABIDOPSIS THALIANA1 (SINAT1), SINAT2, and SINAT6 control autophagosome formation and leaf senescence via controlling stability of the autophagy-related protein ATG13 in Arabidopsis (Qi et al., 2020). U-box E3 ubiquitin ligases PUB12 and PUB13 play multiple functions in immunity, flowering, and senescence. Loss-of-function mutations of PUB12 and PUB13 led to early senescence phenotypes in Arabidopsis under stress conditions (Zhou et al., 2015). BIG BROTHER (BB) also encodes an E3 ligase and is involved in organ size and leaf senescence. Arabidopsis plants overexpressing *BB* displayed early senescence, while *bb* mutants showed delayed senescence phenotypes (Vanhaeren et al., 2017). In rice, dark-induced senescence results in releasing of cytochrome f (Cyt f) from chloroplasts into the cytoplasm, where Cyt f functions to activate caspase-3-like activities by interacting with E3-ubiquitin ligases and RPN9b, the subunit of the ubiquitin proteasome system, ultimately resulting in programmed cell death (PCD) process (Wang et al., 2014).

Phytohormones play very important roles during leaf senescence. ABA, ethylene, jasmonic acid (JA), and salicylic acid (SA) promote senescence, while cytokinins and auxin inhibit this process (Zhang and Guo, 2018; Woo et al., 2019). As a positive regulator of leaf senescence, JA accumulation increases during senescence (He et al., 2002; Hu et al., 2017). Activation of the JA biosynthetic gene *LIPOXYGENASE2* (*LOX2*) by transcription factor TEOSINTE BRANCHED/CYCLOIDEA/PCF4 (TCP4) resulted in early senescence (Suraneni et al., 2010). JA-biosynthetic enzyme 3-ketoacyl-CoA thiolase 2 (KAT2) participates in the catabolism associated with senescence, as well in the early events required for leaf senescence (Castillo and Leon, 2008). As a JA receptor, CORONATINE INSENSITIVE 1 (COI1) is an F-box protein forming complexes with JASMONATE ZIM-domain proteins (JAZ) to mediate their degradation via the 26S proteasome pathway (Xie et al., 1998; Thines et al., 2007; Shan et al., 2011; Kim et al., 2013). The JA-insensitive *coi1-1* mutant displays delayed leaf senescence in *Arabidopsis* (Castillo and Leon, 2008). In the rice (*Oryza sativa*) genome, there are three COI homologs named *OsCOI1a*, *OsCOI1b*, and *OsCOI2*, respectively. *oscoi1b-1* mutant plants displayed delayed leaf senescence under dark and natural conditions. *35S*:*OsCOI1a* or *35S*:*OsCOI1b* could rescue the delayed senescence phenotype of *coi1-1* in *Arabidopsis*, indicating that both OsCOI1a and OsCOI1b play a role in promoting leaf senescence in rice (Lee et al., 2015). JAZs function as negative regulators of the JA signaling pathway via repressing transcriptional activation activities of downstream transcription factors (Chini et al., 2007; Song et al., 2011). JAZ proteins form complex with NOVEL INTERACTOR of JAZs (NINJA)/TOPLESS to restrain JA response by directly regulating various transcription factors (Pauwels et al., 2010; Causier et al., 2012; Kim et al., 2013). JAZ4 and JAZ8 were reported to inhibit JA–induced senescence through interacting with transcription factor WRKY57, which was proposed to play as a balance internode between JA and auxin signaling in regulating senescence (Jiang et al., 2014). JA also interacts with ethylene in regulating senescence in which JAZ proteins interact with ETHYLENE-INSENSITIVE3(EIN3) and ETHYLENE-INSENSITIVE3-LIKE 1(EIL1), two of the key components of the ethylene signaling pathway (Zhu et al., 2011). Furthermore, crosstalk between JA and SA in regulating leaf senescence has been reported. Senescence regulating TF *WRKY53* was found to be antagonistically regulated by JA and SA signals (Miao and Zentgraf, 2007). Among the proteins inhibited by JAZ complexes, MYC2, MYC3, MYC4, and MYC5 displayed accelerated protein degradation under dark or shade conditions but were stabilized in light and after JA treatments (Zhu et al., 2015; Yu et al., 2016). These MYC transcription factors have been shown to be involved in multiple JA response processes via regulating expression of JA responsive genes (Figueroa and Browse, 2012; Zhu et al., 2015; Hu et al., 2017). Recently, It was reported that JA inducible gene *DNA binding-with-one-finger 2.1*(*Dof2.1*) functions in enhancing JA-induced leaf senescence via a MYC2–Dof2.1–MYC2 feed-forward loop (Zhuo et al., 2020).

Dark-induced leaf senescence has been widely studied, in which Ethylene, JA, and Nitric oxide (NO) all play regulatory roles (Fujiki et al., 2001). NO was shown to be involved in regulating dark-induced senescence via ETHYLENE INSENSITIVE 2(EIN2) of the ethylene signaling pathway (Niu and Guo, 2012; Liu and Guo, 2013). It has been shown that endogenous JA content and expression of JA biosynthetic genes increased during dark-induced senescence (Hu et al., 2017; Huang et al., 2017). JAZ7 was reported to regulate dark-induced senescence by interacting with COI1 and MYC2. In *Arabidopsis*, knock-out mutant of *JAZ7* displayed accelerated senescence (Yu et al., 2016). Previous study has demonstrated that MYC2 regulated JA-induced leaf senescence in Arabidopsis by binding to the promoter and activating the expression of SENESCENCE-ASSOCIATED GENE 29(*SAG29*). On the other hand, the bHLH transcription factors (TF) including bHLH03, bHLH13, bHLH14, and bHLH17 repressed the senescence process via antagonistically binding to the promoter and repressing the expression of *SAG29*, resulting in fine-tuned control of JA-induced leaf senescence which assists plants in adapting various environmental changes (Qi et al., 2015). Additionally, ROS plays important roles in JA-induced leaf senescence. JA enhances the accumulation of H_2_O_2_ and reduced H_2_O_2_ content suppresses JA-induced leaf senescence. Results from a recent study revealed that MYC2 was involved in JA-induced H_2_O_2_ accumulation through down-regulating the expression of *CATALASE 2* (*CAT2*) by directly binding to its promoter (Zhang Y. et al., 2020).

Leaf senescence is regulated by a large number of different signals with complex cross-talks among different signaling pathways. Identification and characterization of key senescence regulators that modulate different signaling pathways are therefore of great significance in understanding the molecular mechanisms underlying leaf senescence. In this study, we identified a novel senescence regulator, Ring/U-box protein AtUSR1. Expression of *AtUSR1* was induced by age, darkness, and several plant hormones including JA and ABA. Functional analyses revealed that AtUSR1 plays a positive role in regulating leaf senescence, potentially through regulating the JA signaling pathway. Further study demonstrated that MYC2 can regulate *AtUSR1* expression and AtUSR1 likely functions downstream of the JA-MYC2 signaling pathway in regulating leaf senescence.

## Materials and Methods

### Plant Materials, Growth Conditions, and Stress Treatments

*Arabidopsis thaliana* ecotype Col-0 was used in this study. The *usr1* (*Salk_095353*) and *myc2* [*Salk_017005*, also referred as *jin1-9* (Lorenzo et al., 2004; Jung et al., 2015)] mutant lines were obtained from the Arabidopsis Biological Resource Center (ABRC). Mutants homozygous were obtained by genotyping. Arabidopsis seeds were sterilized in 70% ethanol for 3 min, then washed 3 times by sterile water, and placed on 0.5 × Murashige and Skoog (MS) medium plates, stratified at 4°C for 3 day in the dark, then transferred into continuous light conditions for germination. Plants were grown in soil at 22–24°C under continuous light (100 μmol m^–2^ s^–1^) conditions in a growth room.

For dark treatments of detached leaves, the fifth or sixth leaves from 30-day-old plants were detached and treated in the dark for designated time on filter papers soaked with treatment buffer (3 mM MES, 0.5 × MS, pH 5.8). For dark-induced senescence of attached leaves, fully expanded non-senescence leaves were wrapped with aluminum foil for 6 days.

For hormone treatments, fully expanded non-senescence leaves of 4-week-old plants were detached and immersed in hormone treatment solutions (3 mM MES, 0.5 × MS, pH 5.8) with or without phytohormones (10 μM ABA, 50 μM MeJA, 50 μM IAA, or 10 μM 1-aminocyclopropane-1-carboxylic acid (ACC)) for designated time.

### Generation of Constructs and Transgenic Plants

To generate the *35S*::*AtUSR1* construct, full-length coding sequence (CDS) of *AtUSR1* was polymerase chain reaction (PCR)-amplified and cloned into the pEarleyGate202 (Earley et al., 2006) vector with via gateway cloning methods according to the manufacturer’s instructions (Invitrogen). To obtain the *AtUSR1* complementation construct, *AtUSR1* genomic DNA including the promoter region was PCR-amplified using primers proUSR1-gate-F and AtUSR1gateOER and subcloned into pEarleyGate302 (Earley et al., 2006). To obtain the *proUSR1*::*GUS* construct, the promoter of *AtUSR1* was obtained using primers proUSR1-Glucuronidase (GUS)-F/R, then subcloned into pCAMBIA3301-GUS vector (Abdollahi et al., 2007)at the restriction enzymes digest sites HindIII and NcoI. To generate Dual-luciferase assay constructs, the promoter of *AtUSR1* was amplified using primers proUSR1-pGreenHindIIIF and proUSR1-pGreenBamH1R, then subcloned into the pGreenII 0800-LUC vector at the HindIII and BamH1 sites. Transgenic plants were obtained via the floral-dip method (Clough and Bent, 1998). T3 generation of overexpression plants were used for phenotyping analysis. Primers used in this study are listed in Supplementary Table 1.

### β-Glucuronidase Staining Assay

Histochemical staining was carried out as described previously (Jefferson et al., 1987). Briefly, the sixth leaves of 4-week-old transgenic plants harboring the *proUSR1*::*GUS* construct were incubated in the 5-bromo-4-chloro-3-indolyl-β-D-glucuronide solution (0.5 g/mL of 5-bromo-4-chloro-3-indolyl-β-D-glucuronide, 0.5 mM of potassium ferricyanide, 0.5 mM of potassium ferrocyanide, and 0.1 M of sodium phosphate, pH 7.4) at 37°C for 12 h. Subsequently, the leaves were decolorized in 100% (v/v) ethanol.

### Nitro-Blue Tetrazolium Chloride Staining Assay and H_2_O_2_ Measurement

Nitro-blue tetrazolium (NBT) chloride staining was carried out as previously described (Zhang Z. et al., 2020). Briefly, the fifth leaves of 4-week-old plants were detached and incubated in the NBT staining buffer (0.5 mg/mL NBT in 10 mM potassium phosphate buffer, pH 7.6) overnight, then decolorized in the fixative solution (ethanol: acetic acid: glycerol, 3:1:1) and kept in the ethanol: glycerol (4:1) solution at 4°C. Quantitative H_2_O_2_ measurement was performed according to the methods described previously (Lee et al., 2012).

### Chlorophyll, *Fv/Fm*, and Membrane Leakage Rate Measurement

Chlorophyll was extracted from leaves at different growth stages using 95% ethanol, and chlorophyll content was determined by detecting the absorbance at 665 nm and 649 nm using a UV2400 UV/VIS spectrophotometer as previously described (Zhang and Guo, 2018). The photochemical efficiency of photosystem II (PSII; *Fv/Fm*) was measured using a Chlorophyll Fluorescence Imaging System (Technologica, United Kingdom). Measurements of relative electrolyte leakage were carried out using a bench-top conductivity meter (CON500, CLEAN Instruments). Detached leaves were collected and washed three times using deionized water and immersed in water. Initial conductivity data was collected using the conductor followed by a final conductivity data reading after boiling in 100°C for 10 min to maximum membrane disruption. Total membrane ion leakage was calculated as following: initial conductivity/final conductivity × 100%. Membrane leakage rate was described in the form of percentage of initial conductivity over final conductivity, with 100% ion leakage meaning complete disruption of the membrane system.

### qRT-PCR Analysis

Total RNA was extracted using TRIzol following the manufacturer’s instructions (Invitrogen). The first-strand cDNA was obtained by the Transgene Kit (Transgene Company), which includes the elimination of contaminant genomic DNA. qRT-PCR was carried out using the SYBR Premix Ex Taq (Takara). Each reaction was designed with three technical replicates. Data analysis was done using the 2^–ΔΔCt^ method (Livak and Schmittgen, 2001). The Ct was calculated using the *ACTIN2* gene as an internal control. Three biological replicates were performed for each genotype. Primers used are listed in Supplementary Table 1.

### Dual-Luciferase Assay

For dual-luciferase assay, the pGreenII 0800-LUC vector (Hellens et al., 2000) harboring a firefly luciferase (LUC) gene driven by the *AtUSR1* promoter was generated. The Renilla luciferase (REN) gene driven by the *CaMV35S* promoter was used as an internal control. *35S::AtUSR1* was inserted into the effector plasmid. The reporter and effector plasmids were co-transformed into Arabidopsis mesophyll protoplasts isolated from young and senescence leaves of 4-week–old plants according to the methods described previously (Zhuo et al., 2020). Dual-luciferase assay was performed according to the manufacturer (Promega), Briefly, the luciferase extracted by Passive Lysis buffer (PLB), then value of firefly and REN was obtained in an muti-mode microplate reader (TECAN, Infinite M200 PRO).

### Accession Numbers

The accession numbers for the genes mentioned in this article are listed as follows: *AtUSR1*(At1g14200), *MYC2* (At1g32640), *MYC3*(At5g46760), *MYC4*(At4g17880), *MYC5*(At5g46830), *WRKY57*(At1g69310), *JAZ7*(At2g34600), *SAG12*(At5g45890), *SAG13*(At2g29350), *RBCS3B*(At5g38410), *LOX2*(At3g45140), and *ACTIN2*(At3g18780).

## Results

### Expression of *AtUSR1* Is Associated With Leaf Senescence

**FIGURE 1:**
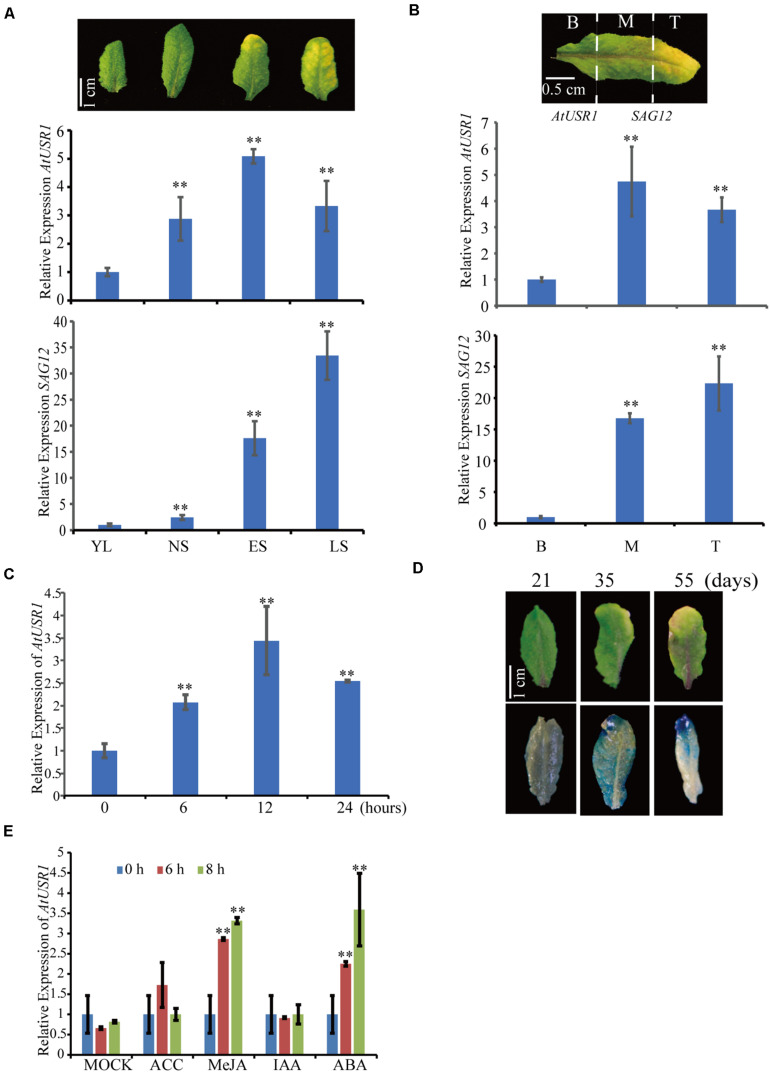
The expression pattern of *AtUSR1*. **(A)** The transcript level of *AtUSR1* was enhanced in an age dependent manner and reached its peak at the ES stage. According to the description of Guo and Gan (2006), gene expression was detected at four different stages of leaf senescence in Arabidopsis. YL, young leaves; NS, fully expanded mature leaves without senescence symptoms; ES, early senescent leaves; and LS, late senescent leaves. The relative values of gene expression (*AtUSR1* and *SAG12*) were calculated based on comparison with gene expression from YL, which was set as 1. **(B)** The expression pattern of *AtUSR1* in indifferent parts of a yellowing leaf; B, Base; M, Middle; and T, Tip. **(C)** The expression pattern of *AtUSR1* under dark conditions. The relative values of gene expression were calculated based on comparison with gene expression from **B**, which was set as 1. **(D)** Histochemical staining of GUS activity detection in leaves at different timepoints as indicated. **(E)** The expression of *AtUSR1* was induced by MeJA and ABA treatments. * and ** indicate significant difference at 0.01 < *P* < 0.05 and *P* < 0.01 levels using student’s *t*-test. Data are shown as the mean ± SD from three independent experiments. Significance analysis was only performed on *AtUSR1* expression.

To identify new regulators of leaf senescence, the GENEVESTIGATOR database^1^ was analyzed and a RING/U-box gene named *AtUSR1* (*U-box Senescence Related 1*, At1g14200), was identified to have enhanced expression in senescing leaves. Results of qRT-PCR analysis showed that *AtUSR1* was significantly up-regulated in senescing leaves (Figure 1A). Within an individual leaf, significant increase of *AtUSR1* expression was observed from the base to the tip where senescence has been initiated (Figure 1B). Expression of SENESCENCE-ASSOCIATED GENE 12 (*SAG12*) was used as an indicator of senescence progress in these analyses. Different from the expression of *SAG12*that was not detected until the last stage of leaf senescence, the expression peak of *AtUSR1* appeared at the early senescence stage. Under darkness, the level of *AtUSR1* transcripts in detached leaves increased gradually and reached a peak at 12 h after dark treatments (Figure 1C).

At different leaf developmental stages, as indicated by GUS staining of *proAtUSR1*::*GUS* transgenic plants, higher promoter activities of *AtUSR1* were detected in yellowing leaves with dark blue staining, whereas less *AtUSR1* promoter activities were detected in younger leaves with light blue staining (Figure 1D).

We further analyzed the effect of phytohormones on the expression of *AtUSR1*. No obvious difference was detected when leaves were treated with ACC or IAA, while significant increase of *AtUSR1* expression was observed when leaves were treated with JA or ABA (Figure 1E).

### Leaf Senescence Is Delayed in the *usr1* Mutant

**FIGURE 2:**
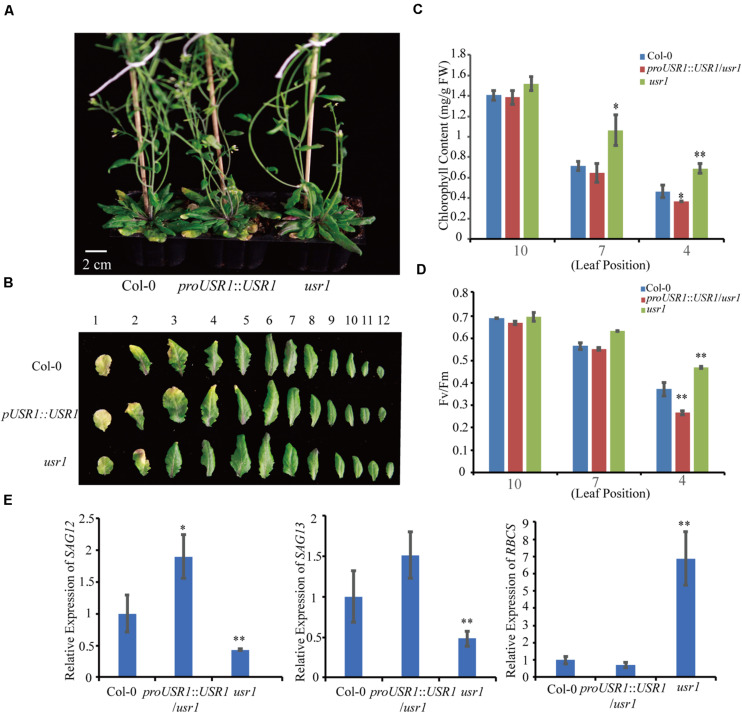
The *usr1* mutant plants show delayed leaf senescence. **(A)** The senescence phenotype of plants with different genotype as indicated after grown under continuously light for 40 days. *usr1* mutant shows delayed leaf senescence and complementation plants *proUSR1*::*USR1*/*usr1* rescued the delayed leaf senescence phenotype of *usr1* plants. **(B)** Senescence phenotypes of detached 1st–12th leaves of different genotypes as indicated. **(C,D)** Chlorophyll content and *Fv/Fm* detection of leaves from different leaf positions in Col-0, *usr1*, and *proUSR1*::*USR1*/*usr1* plants as indicated. **(E)** Expression of *SAGs* genes including *SAG12*, *SAG13*, and *RBCS* in plants of different genotypes as indicated. Single and double asterisk indicate significant difference at 0.01 < *P* < 0.05 and *P* < 0.01 levels using student’s *t*-test. Data are shown as the mean ± SD from three independent experiments.

To investigate the function of AtUSR1 in leaf senescence, we obtained an AtUSR1 knock-down mutant (*usr1*) in which a T-DNA fragment is inserted in the 5′UTR region of this gene (Supplementary Figure 1A). The *AtUSR1* expression level was significantly lower in *usr1* than in WT Col-0 (Supplementary Figure 1B). A complementation assay was carried out in which the full length genomic DNA of *AtUSR1* (*proAtUSR1*::*AtUSR1*) was used to transform the *usr1* mutant to generate complementation plants. Under our growth conditions, older leaves from 6-week-old Col-0 plants started to exhibit visible yellowing, while the counterpart leaves from *usr1* plants were still green. The complementation plants displayed similar senescence progress to Col-0 (Figures 2A,B), indicating that the *proUSR1*::*AtUSR1* transgene was able to rescue the delayed senescence phenotype of *usr1* plants.

Total chlorophyll contents of the 4th and 7th leaves of *usr1* plants were significantly higher than that of Col-0 (Figure 2C). As an indicator of photosynthetic efficiency, the *Fv/Fm* ratio in *usr1* leaves was significantly higher than Col-0 (Figure 2D). Meanwhile, the complementation plants displayed similar chlorophyll contents and *Fv/Fm* values to Col-0 (Figures 2C,D). Similarly, the *usr1* mutant displayed lower transcripts of the senescence marker genes *SAG12* and *SAG13* but higher expression of the photosynthetic gene *RBCS* compared with Col-0 and the complementation plants (Figure 2E).

In this study, *proUSR1::USR1/usr1* transgenic plants displayed an slightly early senescence phenotype compared with WT (Figure 2). One possibility is that multiple copies of the T-DNA insertion led to higher expression of *AtUSR1* than WT (Supplementary Figure 1).

### Overexpression of *AtUSR1* Accelerates Leaf Senescence

**FIGURE 3:**
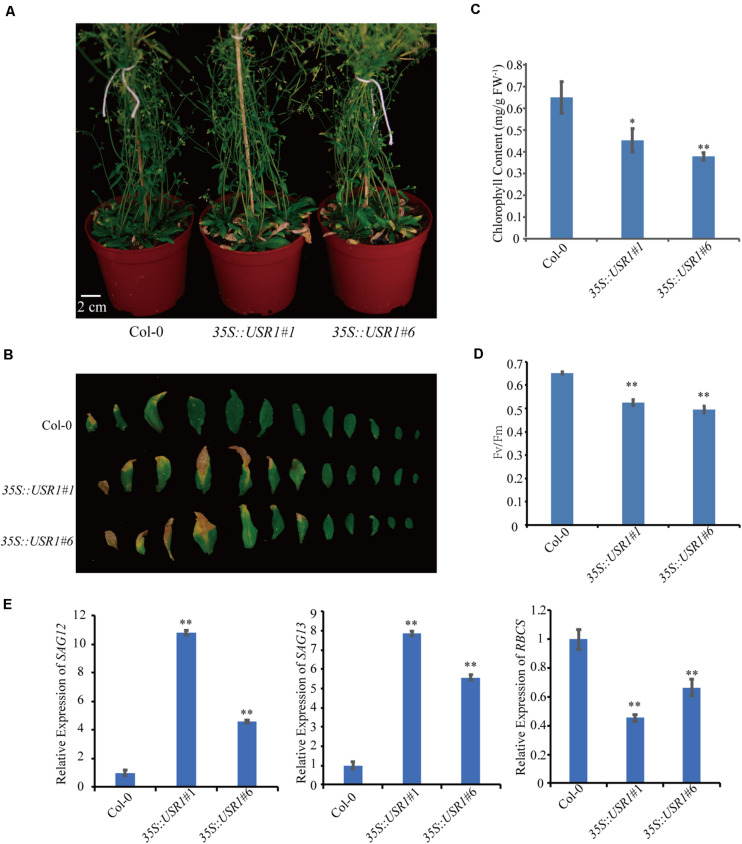
*AtUSR1* overexpression accelerates leaf senescence. **(A)** The precocious senescence phenotype of *AtUSR1* overexpression lines after grown under continuously light condition for 40 days. **(B)** The phenotype of 1st–12th leaves detached from plants with different genotypes. **(C,D)** Chlorophyll content and *Fv/Fm* detection at different leaf positions in Col-0, two independent *AtUSR1* overexpression plants as indicated. **(E)** Expression pattern of *SAG12*, *SAG13*, and *RBCS* in plants overexpressing *AtUSR1* and in Col-0. Single and double asterisk indicate significant difference at 0.01 < *P* < 0.05 and *P* < 0.01 levels using student’s *t*-test. Data are shown as the mean ± SD from three independent experiments.

To further understand the role of AtUSR1 in leaf senescence, transgenic plants harboring *35S*::*AtUSR1* were generated and two representative lines (*35S*::*AtUSR1#1, 35S*::*AtUSR1#6*) were used for further characterization (Supplementary Figure 2). Under continuous light, 5-week-old plants of *35S*::*AtUSR1#1* and *35S*::*AtUSR1#6* displayed a premature leaf senescence phenotype compared with Col-0 (Figures 3A,B). Consistent with the visible phenotype, chlorophyll levels, and *Fv/Fm* ratios of both *AtUSR1* overexpression lines were lower than Col-0 (Figures 3C,D). The expression of *SAG12* and *SAG13* was significantly increased while the expression of *RBCS* was reduced in *AtUSR1* overexpression plants compared with Col-0 (Figure 3E). Together, these results suggested that AtUSR1 acted as a positive regulator of leaf senescence.

### AtUSR1 Promotes Dark-Induced Senescence

**FIGURE 4:**
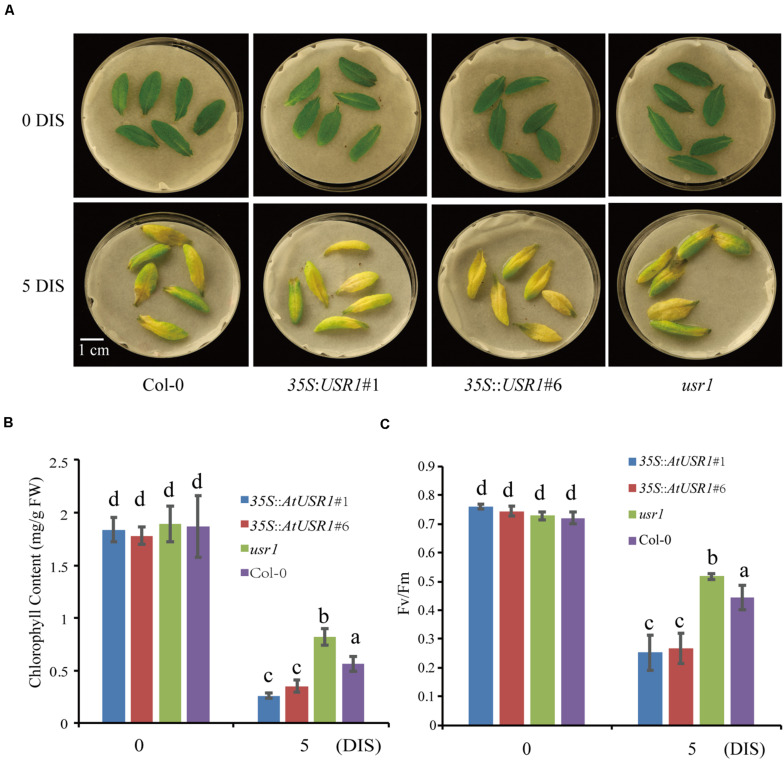
AtUSR1 is involved in dark-induced leaf senescence. **(A)** *AtUSR1* Overexpression lines displayed precocious leaf senescence while the *usr1* mutant showed delayed senescence under dark conditions. **(B,C)** Total chlorophyll content and *Fv/Fm* of different genotypes as indicated. The data in **(B,C)** different letters above columns indicate significant differences according to Duncan’s multiple range test (*P* < 0.05). Data are shown as the mean ± SD from three independent experiments.

Since *AtUSR1* expression was induced by both senescence and darkness, we studied the role of AtUSR1 in dark-induced senescence. After incubated under dark conditions for 5 days, detached leaves of the *usr1* mutant retained green color while the *35S*::*AtUSR1*#1 and *35S*::*AtUSR1*#6 leaves showed accelerated leaf yellowing compared to that of Col-0 (Figure 4A). Total chlorophyll levels and *Fv/Fm* ratios of the *AtUSR1*-overexpressing lines were significantly lower than the *usr1* mutant after the dark treatment (Figures 4B,C).

We also studied dark-induced senescence of attached leaves from Col-0, *AtUSR1* overexpression, and *usr1* plants. Fully expanded non-senescence leaves were wrapped with aluminum foil for dark treatments. 6 days after treatments, the wrapped leaves of *usr1* plants stayed green while dark-treated leaves of the *35S*::*AtUSR1* lines displayed senescence symptoms compared with Col-0 (Supplementary Figure 2A). Compared to Col-0, chlorophyll content was higher in leaves of *usr1* and lower in *35S*::*AtUSR1* leaves while membrane leakage rates were lower in *usr1* leaves and higher in *35S*::*AtUSR1* leaves (Supplementary Figures 2B,C). The above described results suggested that AtUSR1 positively regulates dark-induced senescence as well.

### AtUSR1 Affects ROS Homeostasis

**FIGURE 5:**
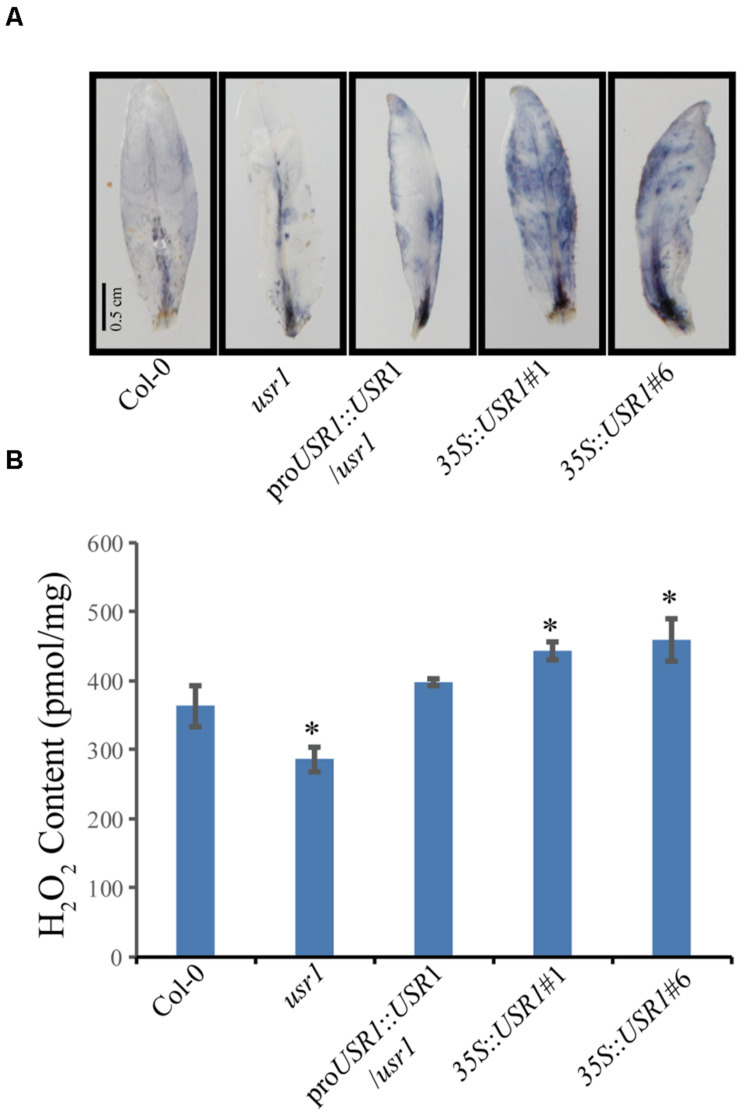
AtUSR1 affects ROS accumulation. **(A)** The NBT staining results of leaves from different genotypes. **(B)** Accumulation of H_2_O_2_ in detached leaves of different genotypes as indicated. *Indicate significant difference at 0.01 < *P* < 0.05 levels using student’s *t*-test. Data are shown as the mean ± SD from three independent experiments.

Reactive oxygen species play important roles in senescence as well as stress responses (Sedigheh et al., 2011; Jajic et al., 2015). Cellular levels of ROS in fully expanded non-senescence leaves with different genotypes were measured by NBT staining and H_2_O_2_ quantification. The results showed that leaves of the *35S*::*AtUSR1* lines displayed stronger while the *usr1* mutant showed weaker NBT staining compared with Col-0, suggesting that AtUSR1 rendered plant accumulating more ROS in leaves (Figure 5A). Leaves from *35S*::*AtUSR1*#1 to *35S*::*AtUSR1*#6 lines contained significantly higher amount of H_2_O_2_ while the *usr1* mutant had lower levels of H_2_O_2_ accumulation compared with Col-0 (Figure 5B).

### AtUSR1 Is Involved in JA-Mediated Senescence

**FIGURE 6:**
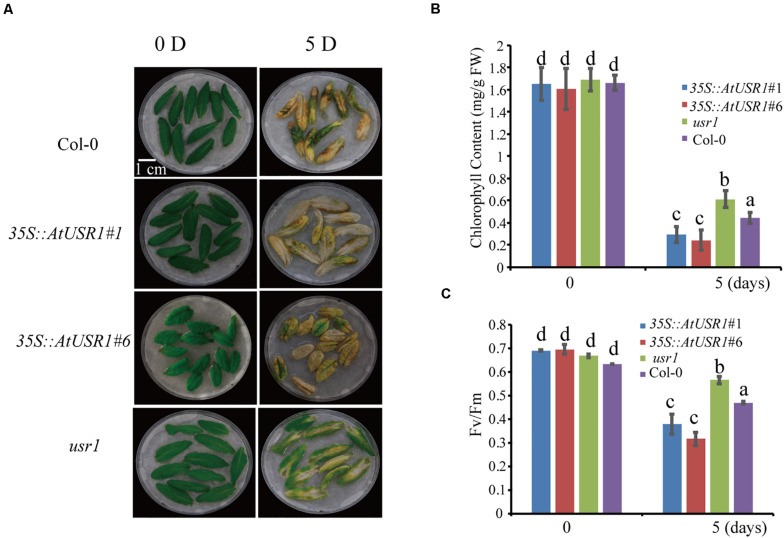
AtUSR1 plays as positive role in JA mediated leaf senescence. **(A)** *AtUSR1* overexpression enhanced JA-induced leaf senescence while *usr1* mutation delayed this process. Detached Sixth leaves of different genotypes were treated with 50 μM MeJA for 5 days. **(B,C)** Total chlorophyll content and *Fv/Fm* in the leaf samples of different genotypes as indicated. The data in **(B,C)** different letters above columns indicate significant differences according to Duncan’s multiple range test (*P* < 0.05). Data are shown as the mean ± SD from three independent experiments.

Jasmonic acid is a senescence-promoting signal in both age-dependent and dark-induced senescence (Yu et al., 2016; Hu et al., 2017). Our data indicated that *AtUSR1* expression was up-regulated by JA treatments (Figure 1E). We therefore tested the role of AtUSR1 in JA-induced senescence. After treated with exogenous MeJA for 5 days, detached leaves from *35S*::*AtUSR1* plants were completely yellow, while *usr1* leaves remained mostly green and leaves of Col-0 were in-between (Figure 6A). Chlorophyll content and *Fv/Fm* ratio data also indicated that the *usr1* mutation delayed while *AtUSR1* overexpression accelerated JA-induced senescence on detached leaves (Figures 6B,C).

### AtUSR1 Functions Downstream to MYC2 in Regulating Leaf Senescence

Previous studies have demonstrated that MYC2 plays an essential role in JA-induced senescence (Yu et al., 2016). Transcription factor MYC2 binds to the G-box motif (CACGTG) and its variants such as E-box (CANNTG), G/A box (CACGAG), and G/C box (CACGCG) to regulate expression of target genes (Dombrecht et al., 2007; Zhuo et al., 2020). Interestingly, using PLACE^2^, we have identified a E-box [CAN(A)N(T)TG] motif in the promoter region of *AtUSR1* (−130 bp to −124 bp upstream of the translation start site).

**FIGURE 7:**
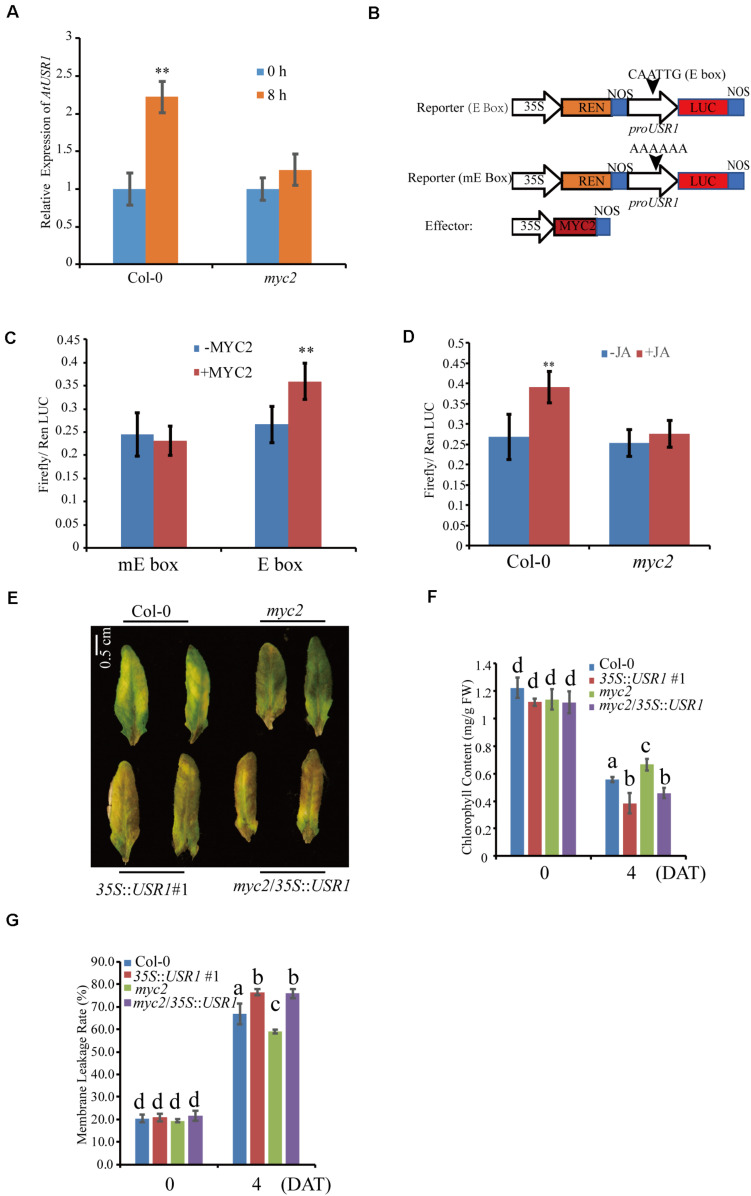
AtUSR1 functions downstream to MYC2. **(A)** The expression pattern of *AtUSR1* in Col-0 and the *myc2* mutant under 100 μM MeJA treatments. **(B)** Schematic diagram of the reporter and effector constructs used in the dual luciferase assay. **(C)** MYC2 increased the activities of the *AtUSR1* promoter. The promoter of *AtUSR1* containing an E-box (CAGCGT) or a mutant version of the E-box (AAAAAA) fused with LUC was co-transformed with an effecter construct with or without *35S*::*MYC2* into Col-0 protoplasts. Ren LUC activity acted as internal control. The experiments presented here were done only with Col-0 plants (labeled as –MYC2). **(D)** MeJA induced the expression of *AtUSR1* and this induction was MYC2-dependent. The reporter construct (*proAtUSR1*::*LUC*) was transformed into protoplasts of Col-0 or *myc2*, respectively. Ren Luc acted as an internal control. **(E)** The cross line myc2/35S::USR1 rescued the delayed senescence phenotype of *myc2* under MeJA treatments. **(F)** Chlorophyll contents in different genotypes as indicated. **(G)** Membrane leakage rates in different genotypes as indicated. **Indicate significant difference at *P* < 0.01 levels using student’s *t*-test. Data are shown as the mean ± SD from three independent experiments. The data in **(F,G)** different letters above columns indicate significant differences according to Duncan’s multiple range test (*P* < 0.05).

We then examined the expression of *AtUSR1* in the *myc2* mutant under MeJA treatments. Detached fully expanded leaves were treated with MeJA for 8 h, after which *AtUSR1* expression was observed to be significantly increased in Col-0 but this increase was significantly reduced in the *myc2* mutant (Figure 7A), suggesting that the JA-induction of *AtUSR1* expression is partially MYC2-dependent.

We further analyzed the MYC2 regulation of *AtUSR1* expression via a dual-luciferase assay. In the reporter construct, the *Firefly luciferase* gene was driven by the *AtUSR1* promoter containing the E-box sequence and the REN gene was used as an internal reference (Figure 7B). The reporter construct was used in co-transfecting of Col-0 protoplasts together with an effector construct in which *MYC2* was under the control of the 35S promoter. The ratio of Firefly/Renilla activities was significantly higher when the effector construct harboring *35S::MYC2* was co-transformed with the reporter construct containing the *AtUSR1* promoter. The increase in Firefly/Renilla ratio was not detected when the E-box on the *AtUSR1* promoter was replaced by a mutated sequence (Figure 7C). These results suggest that MYC2 could regulate the expression of *AtUSR1* and this regulation is dependent on the E-box on its promoter.

Next, we studied the role of MYC2 in JA-regulated expression of *AtUSR1* using the same dual luciferase strategy. The *35S*::*MYC2* effector construct was co-transfected with a reporter construct in which firefly LUC was driven by the *AtUSR1* promoter. In the same reporter construct, Renilla LUC driven by the *35S* promoter was used as an internal control. This combination was co-transfected into Col-0 protoplasts with or without JA treatments. The results indicated that Firefly/Renilla was significantly higher under JA treatments compared with the mock. The increase of Firefly/Renilla caused by JA treatments disappeared when *myc2* mutant protoplasts were transfected, suggesting that the MeJA induction of *AtUSR1* promoter activities was affected in the *myc2* mutant (Figure 7D).

Mutation of *MYC2* has been reported to delay JA-induced chlorophyll degradation (Zhu et al., 2015). To further study the relationship between *AtUSR1* and MYC2 in JA-induced senescence, we obtained *myc2* mutant plants harboring *35S*::*AtUSR1* (referred to as *myc2*/*35S*::*AtUSR1*) and senescence phenotypes of detached leaves were examined in presence of MeJA. Similar to *35S*::*AtUSR1* leaves that displayed early senescence phenotypes compared to Col-0, leaves of *myc2*/*AtUSR1OE* plants also showed precocious senescence, rescuing the delayed senescence phenotype of the *myc2* mutant (Figure 7E). Moreover, the effects of *myc2* mutation on chlorophyll content and *Fv/Fm* of MeJA-treated leaves were reduced by *AtUSR1* overexpression (Figures 7F,G). The above-described results suggested that AtUSR1 acted downstream to MYC2 in mediating JA-induced senescence.

## Discussion

As the last stage of development, leaf senescence is a crucial process that influences photosynthetic capacity and reallocation of nutrients from senescing leaves into young leaves and reproductive organs (Ahmad and Guo, 2019; Krieger-Liszkay et al., 2019). Timely senescence is essential for plants’ reproductive success. Precocious or early senescence caused by harsh growth conditions, on the other hand, compromises crop yield in an agricultural setting (Wu et al., 2012; Bengoa Luoni et al., 2019). Initiation and progression of leaf senescence can be affected by a large number of endogenous and environmental factors including developmental stage, age, phytohormones, environmental cues such as temperature, darkness, and pathogens (Bresson et al., 2018). All these factors and related signaling pathways form a complex network in fine-tuning the initiation and progression of leaf senescence. Identifying senescence-regulating genes and clarifying their functions are of great importance in devising genetic strategies of manipulating senescence for improving crop yield and production traits (Jing and Nam, 2012).

A large number of genes undergo substantial changes in expression during senescence, including genes related to protein degradation, nutrient remobilization, chlorophyll metabolism, and transcription regulation (Sato et al., 2009; Goud and Kachole, 2011; Izumi and Ishida, 2011; Sakuraba et al., 2012; Tamary et al., 2019). Also included are regulators involved in signaling transduction in response to phytohormones such as ethylene, JA, ABA, auxin, and cytokinins (Jan et al., 2019).

Here we report the function of a ring/u-box protein AtUSR1 in promoting leaf senescence mediated by the JA-MYC2 pathway. Firstly, the expression of *AtUSR1* was age-dependent and was induced by darkness, JA, and ABA treatments (Figure 1). Interestingly, different from the expression of *SAG12* that was not detected until the last stage of leaf senescence, the expression peak of *AtUSR1* appeared at the early senescence stage indicating that *AtUSR1* plays potential roles in the initial of senescence stage. Leaves of *usr1* plants showed an obvious delayed senescence phenotype and *proAtUSR1*::*AtUSR1* was able to rescue the mutant phenotype (Figure 2). Overexpression of *AtUSR1* caused early leaf senescence (Figure 3). In addition, results from dark treatments of *usr1* and *35S*::*AtUSR1* plants indicated that AtUSR1 functioned in accelerating dark-induced senescence as well (Figure 4). Further study demonstrated that *AtUSR1*overexpression promoted JA-induced senescence and knocking-down of *AtUSR1* delayed this process (Figure 6).

In this study, we demonstrated that AtUSR1 acts downstream of MYC2 in JA-induced leaf senescence. Firstly, MeJA treatments enhanced *AtUSR1* expression in a MYC2-dependent manner (Figure 7A). Results of dual luciferase assays indicated that MYC2 could regulate *AtUSR1* expression (Figures 7C,D). In the presence of JA, JAZ proteins undergo degradation via ubiquitin pathways mediated by the F-box protein COI1. MYC2 is thus released from the JAZ-MYC2 complex and function to activate the expression of downstream genes including *AtUSR1*, that functions in promoting leaf senescence (Figure 7). In the absence of JA, the JAZ-MYC2 complexes inhibit the activity of MYC2 and *AtUSR1* transcription is compromised.

We also found that AtUSR1 affects ROS homeostasis. ROS over-accumulated in *35S*::*AtUSR1* leaves and the *usr1* mutant had less ROS (Figure 5). ROS are known to be involved in multiple biological processes in plants such as leaf senescence, stress response, and hypersensitive response (Sedigheh et al., 2011; Jajic et al., 2015). Since ROS can interplay with other signaling molecules in regulating plant development and stress responses (Hoque et al., 2012; Li et al., 2018), it’s reasonable to hypothesize that AtUSR1 could also be involved in multiple signaling pathways. Significant overlap of gene expression changes exists between natural senescence, treatments of senescence-promoting phytohormones and stress conditions (Guo, 2013). AtUSR1 can potentially function as an internode factor that can be affected by multiple senescence-promoting signaling pathways induced by age, darkness, and JA.

## Data Availability Statement

The original contributions presented in the study are included in the article/Supplementary Material, further inquiries can be directed to the corresponding author/s.

## Author Contributions

YG conceived the project. ZZ and YG designed the research, performed data analysis, and wrote the manuscript. ZZ and MX performed experiments. All authors contributed to the article and approved the submitted version.

## Conflict of Interest

The authors declare that the research was conducted in the absence of any commercial or financial relationships that could be construed as a potential conflict of interest.

## Footnotes

1 https://www.genevestigator.com/gv/index.jsp

2 https://www.dna.affrc.go.jp/PLACE/?action=newplace

## Supplementary Material

Supplementary Figure 1 Genotyping of the *usr1* mutant. **(A)** The Gene structure of *AtUSR1* and the *usr1* (*Salk_095353*) T-DNA insertion in the 5′UTR region. **(B)** Transcript levels of *AtUSR1 in Col-0, usr1* mutant, and complementation plants harboring *proAtUSR1::USR1/usr1*.

Supplementary Figure 2 Expression of *AtUSR1* in two independent overexpression lines.

Supplementary Figure 3 Phenotype of attached leaves under dark conditions. **(A)** Fully expanded leaves were wrapped with aluminum foil for 6 days. **(B)** Chlorophyll contents of different genotypes as indicated. **(C)** Membrane leakage rates of different genotypes as indicated.

Supplementary Table 1 Primers used in this study.
