# Supplementary figures and images for: Ring/U-Box Protein AtUSR1 Functions in Promoting Leaf Senescence Through JA Signaling Pathway in Arabidopsis
Source: Front Plant Sci. 2020 Dec 16;11:608589. doi: 10.3389/fpls.2020.608589 (PMC7772223; doi:10.3389/fpls.2020.608589)

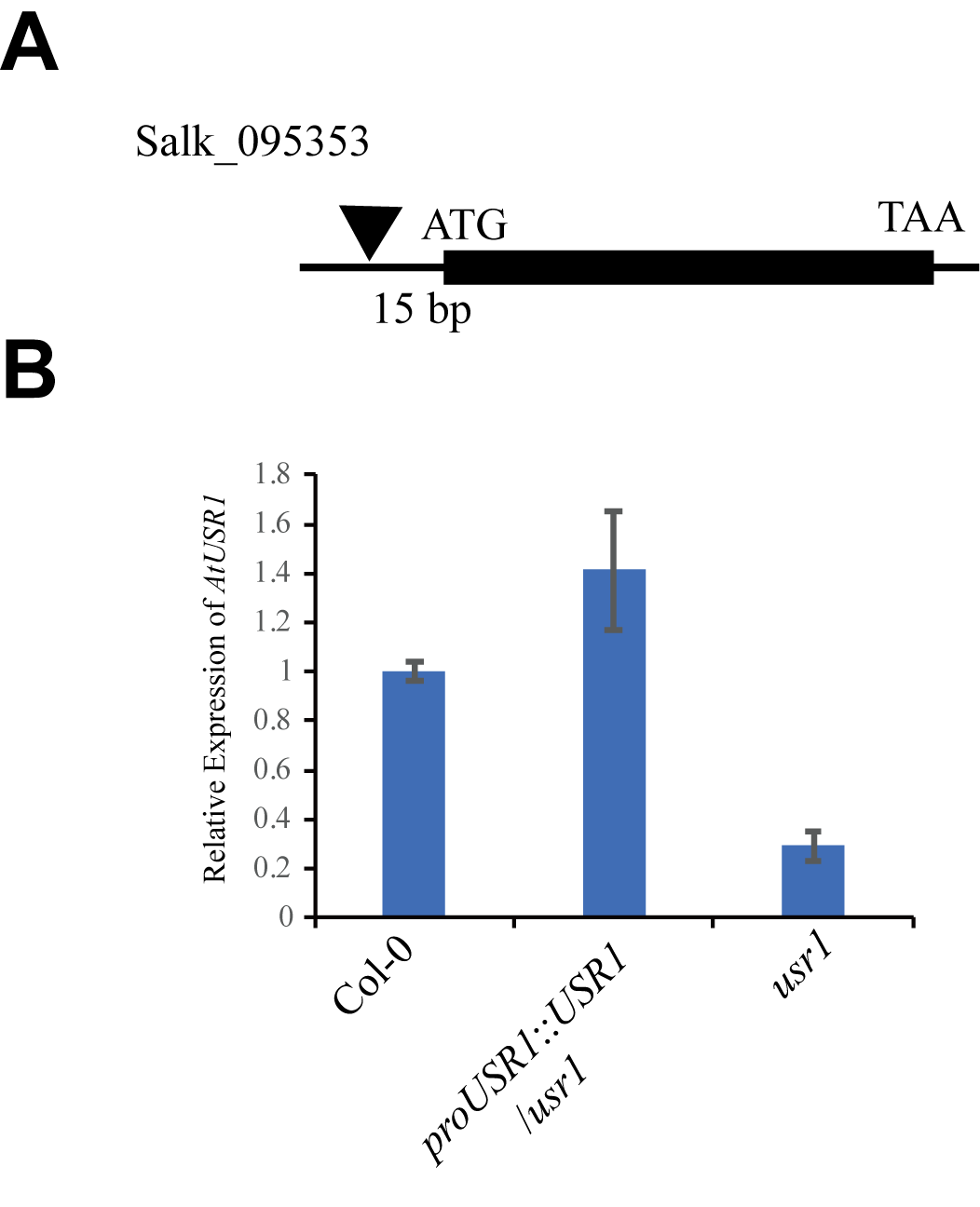

Supplement: Supplementary Figure 1 — Genotyping of the usr1 mutant. (A) The Gene structure of AtUSR1 and the usr1 (Salk_095353) T-DNA insertion in the 5′UTR region. (B) Transcript levels of AtUSR1 in Col-0, usr1 mutant, and complementation plants harboring proAtUSR1::USR1/usr1. [file Image_1.TIF]

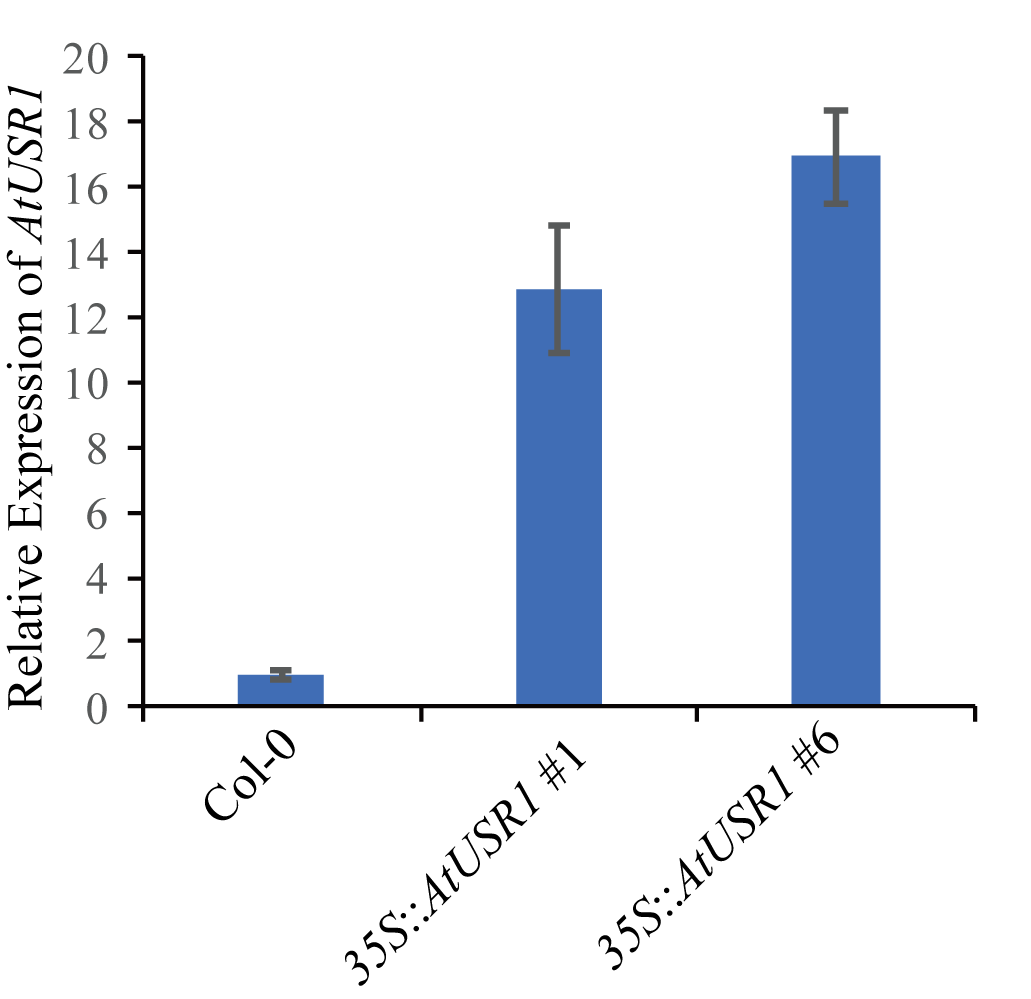

Supplement: Supplementary Figure 2 — Expression of AtUSR1 in two independent overexpression lines. [file Image_2.TIF]

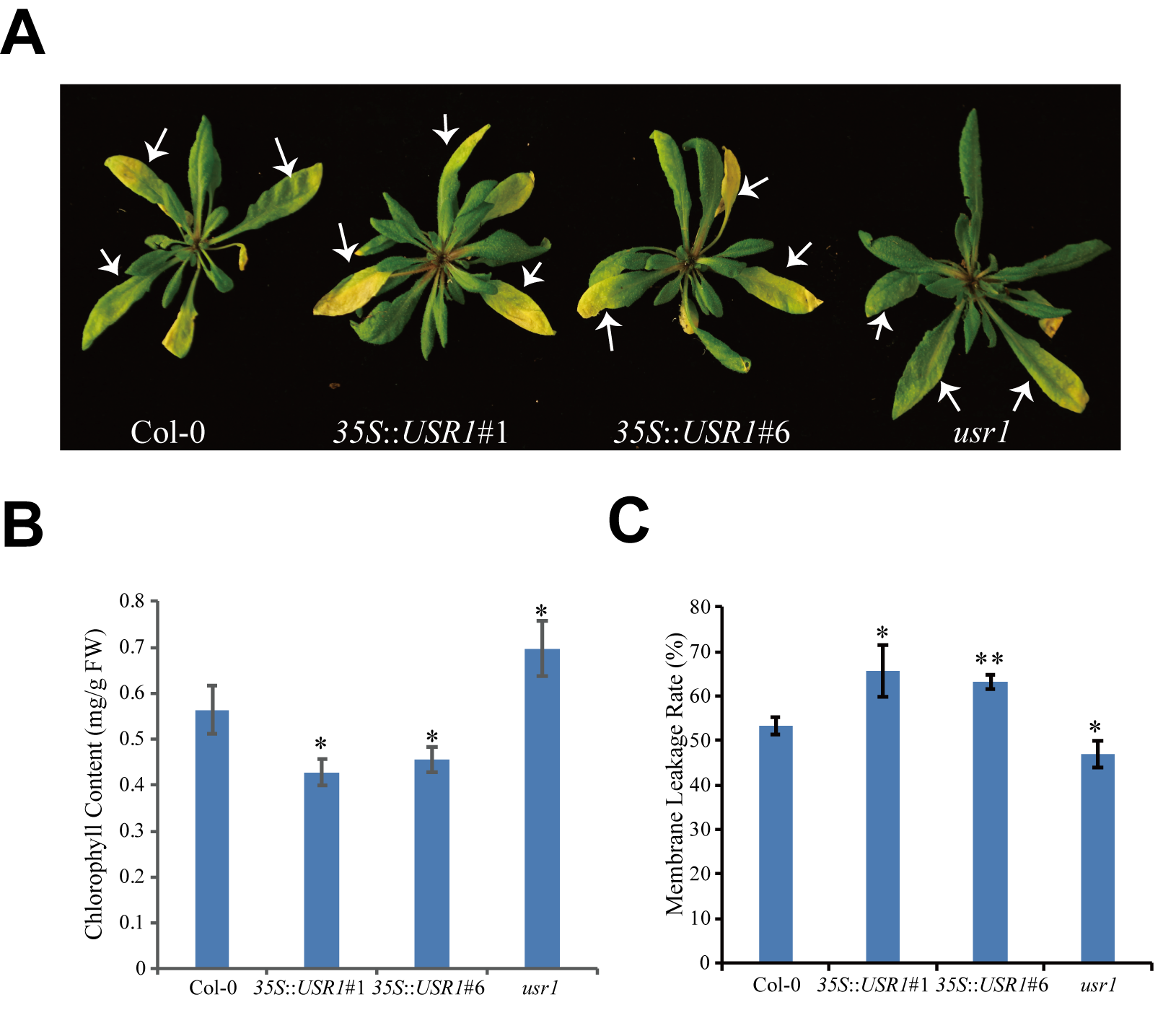

Supplement: Supplementary Figure 3 — Phenotype of attached leaves under dark conditions. (A) Fully expanded leaves were wrapped with aluminum foil for 6 days. (B) Chlorophyll contents of different genotypes as indicated. (C) Membrane leakage rates of different genotypes as indicated. [file Image_3.TIF]
