# Supplementary material for: Ring/U-Box Protein AtUSR1 Functions in Promoting Leaf Senescence Through JA Signaling Pathway in Arabidopsis
Source: Front Plant Sci. 2020 Dec 16;11:608589. doi: 10.3389/fpls.2020.608589 (PMC7772223; doi:10.3389/fpls.2020.608589)
Supplement: Supplementary Table 1 — Primers used in this study. [file Table_1.docx]

**Supplementary Table 1. Primers Used in This Study**

| Primers Name | Primers Sequence (5'-3') |
| --- | --- |
| Q_SAG12_F | TCCAATTCTATTCGTCTGGTGTGT |
| Q_SAG12_R | CCACTTTCTCCCCATTTTGTTC |
| Q_ACT2_F | TGTGCCAATCTACGAGGGTTT |
| Q_ACT2_R | TTTCCCGCTCTGCTGTTGT |
| Q_RBCS3B_F | AGTAATGGCTTCCTCTATGC |
| Q_RBCS3B_R | GTGATGTCCTTGTTGGTCTTG |
| USR1-QF | TCAAAGTGTGTGGAGGAGTGGT |
| USR1-QR | TCGCCGGCGTTAATGGAGAA |
| AtUSR1gateOEF: | GTGGGGACAAGTTTGTACAAAAAAGCAGGCTTCATGTCTTCTGAGAATGATTTC |
| AtUSR1gateOER: | GTGGGGACCACTTTGTACAAGAAAGCTGGGTCGTTTCTTCTGTCGCCGGCGTTA |
| proUSR1-gate-F: | GTGGGGACAAGTTTGTACAAAAAAGCAGGCTTCGAATTTAATCAAACACCTTCTG |
| proUSR1-GUS-F | GCAGGCATGCAAGCTTCATCGAAAGTCAGAACATTACC |
| proUSR1-GUS-R | CTCAGATCTACCATGGCACGCTAGTACCCAAAACAAAG |
| proUSR1-pGreenHindIIIF | CGGTATCGATAAGCTTGAATTTAATCAAACACCTT |
| proUSR1- pGreenBamH1R | TAGAACTAGTGGATCCCACGCTAGTACCCAAAACAAA |
| usr1-genotypingLP | CCTCCTGTTCCTAGTTTTCGG |
| usr1-genotypingRP | ACCACTCCTCCACACACTTTG |
